# Novel Betanucleorhabdoviruses Infecting Elderberry (*Sambucus nigra* L.): Genome Characterization and Genetic Variability

**DOI:** 10.3390/pathogens13060445

**Published:** 2024-05-24

**Authors:** Dana Šafářová, Thierry Candresse, Jana Veselská, Milan Navrátil

**Affiliations:** 1 Department of Cell Biology and Genetics, Faculty of Science, Palacký University, Olomouc, Šlechtitelů 27, 783 71 Olomouc-Holice, Czech Republic; jana.veselska@upol.cz (J.V.); milan.navratil@upol.cz (M.N.); 2 UMR 1332 Biologie du Fruit et Pathologie, INRA, University of Bordeaux, 33140 Villenave d’Ornon, France; thierry.candresse@inrae.fr

**Keywords:** high-throughput sequencing, plant rhabdovirus, genetic diversity, mixed infection

## Abstract

The genus *Betanucleorhabdovirus* includes plant viruses with negative sense, non-segmented, single-stranded RNA genomes. Here, we characterized putative novel betanucleorhabdoviruses infecting a medically important plant, elderberry. Total RNA was purified from the leaves of several plants, ribodepleted and sequenced using the Illumina platform. Sequence data analysis led to the identification of thirteen contigs of approximately 13.5 kb, showing a genome structure (3′-N-P-P3-M-G-L-5′) typical of plant rhabdoviruses. The detected isolates showed 69.4 to 98.9% pairwise nucleotide identity and had the highest identity among known viruses (64.7–65.9%) with tomato betanucleorhabdovirus 2. A detailed similarity analysis and a phylogenetic analysis allowed us to discriminate the elderberry isolates into five groups, each meeting the sequence-based ICTV demarcation criterion in the *Betanucleorhabdovirus* genus (lower than 75% identity for the complete genome). Hence, the detected viruses appear to represent five novel, closely related betanucleorhabdoviruses, tentatively named Sambucus betanucleorhabdovirus 1 to 5.

## 1. Introduction

The genus *Betanucleorhabdovirus*, together with *Alphanucleorhabdovirus* and *Gammanucleorhabdovirus*, is one of the three recently established genera of plant nucleorhabdoviruses [1,2,3], which are classified within the subfamily *Betanucleorhabdovirinae*, family *Rhabdoviridae*. Like all nucleorhabdoviruses, its members have a negative-sense, non-segmented, single-stranded RNA genome of 13.2–15.2 kb in length. This genome contains six typical rhabdovirus genes, in order 3′-N-P-P3-M-G-L-5′, separated by junction sequences with a conserved motif and encoding the various viral proteins: nucleocapsid protein (N), phosphoprotein (P), putative movement protein (P3), matrix protein (M), glycoprotein (G) and the large protein/polymerase (L). The genomic RNA is encapsidated in bacilliform virions 45–100 nm in diameter and 130–300 nm long. The virus replicates in the plant nuclei and in the insect vector, and virions are formed in the perinuclear space [2,4]. Betanucleorhabdoviruses infect dicots, mainly herbaceous but also woody plants; however, each of them has a rather restricted host range. They are transmitted by aphids in a persistent manner [1,4,5]; mechanical transmission is also possible but has probably no impact on virus spread in nature and is mainly used for experimental purposes [4]. Seed or pollen transmission was not described until now [1,2,4].

In agreement with the current recommendations of the International Committee on the Taxonomy of Viruses (ICTVs) [2,6], the genus *Betanucleorhabdovirus* currently comprises 18 members described based on the primary observation of the symptoms of infection and on viral genome sequencing and, more recently, on the increasing availability of high-throughput sequencing, as latent infections of plants and from plant transcriptome data. Currently accepted *Betanucleorhabdovirus* species include *Betanucleorhabdovirus asclepiadis* (represented by Asclepias syriaca virus 2, AscSyV2), *B. bacopae* (Bacopa monnieri virus 2, BmV2) [7], *B. cardamomi* (cardamom vein clearing virus, CdCV) [8], *B. cnidii* (Cnidium virus 1, CnV1) [5], *B. daturae* (Datura yellow vein virus, DYVV) [9], *B. loti* (bird’s-foot trefoil-associated virus, BFTV) [10], *B. mali* (apple rootstock virus A, ApRVA) [11], *B. medicagonis* (alfalfa-associated nucleorhabdovirus, AaNV) [12], *B. plectranthi* (Plectranthus aromaticus virus 1, PleArV1) [13], *B. retesonchi* (Sonchus yellow net virus, SYNV) [14], *B. rhododendri* (Rhododendron delavayi virus 1, RhoDeV1) [1], *B. ribes* (blackcurrant-associated rhabdovirus, BCaRV) [15], *B. venasonchi* (sowthistle yellow vein virus, SYVV) [16], *B. zanthoxyli* (Zhuye pepper nucleorhabdovirus, ZPNRV) [17]. Newly added genus members include the following: *B. alphalycopersici* (tomato betanucleorhabdovirus 1, TBRV1), *B. betalycopersici* (tomato betanucleorhabdovirus 2, TBRV2), *B. taraxi* (Taraxacum betanucleorhabdovirus 1, TarBRV1) and *B. picridis* (Picris betanucleorhabdovirus 1, PBRV1) [13,18]. But betanucleorhabdoviruses are being detected and described as infecting other plants, such as Cuscuta reflexa virus 1, Persicaria minor virus 1 [1] and Paris yunnanensis betanucleorhabdovirus 1 [19]. Despite this list, the number of ssRNA(−) plant viruses and information about them is still quite limited compared to ssRNA(+) viruses [1].

Elderberry (*Sambucus nigra* L., *Sambucus nigra* subsp. *canadensis*) is usually a deciduous wild shrub or, in the case of cultivars grown in production orchards, a small tree commonly found in the temperate zone [20,21]. It is very popular in traditional medicine due to the antiviral, antibacterial and anti-inflammatory effect of extracts from elderberry fruits and flowers. It also has other biopharmaceutical properties such as antidiabetic, antitumor, and antidepressant effects, and impacts obesity and metabolic dysfunctions, all connected mainly with the antioxidants and polyphenols present in the fruits and flowers. This has made the cultivation of elderberry and elderberry fruit production very attractive in pharmaceutical research and for its use in medical practice [22,23,24,25,26,27].

The present study reports the characterization of novel nucleorhabdoviruses detected in elderberry, and their molecular and biological characterization.

## 2. Materials and Methods

### 2.1. Plant Material

As part of an elderberry virome survey, the wild growing elderberry shrub (designated B15) was sampled in 2015 at the Slatinky site, Czech Republic (GPS 49.5405722 N, 17.0919633 E) and a high-throughput sequencing was performed on its purifed dsRNA. Due to the presence of several virus isolates of different species, it was not possible to assemble the complete genome of putative betanucleorhabdovirus isolates from the sequence data thus obtained. Therefore, other elderberry materials were screened using the RhabF and RhabR primer pair (see below) designed from the partial contigs obtained from the B15 plant. Three elderberry plants from three different localities were thus selected in 2020 for further analysis; two of them, B160 collected from Slatinky (in close vicinity of the original B15 shrub) and B42 from Olomouc (49.5777133 N, 17.2787939 E), both in Central Moravia, are wild growing shrubs, and the third one (B78) is a tree of cv. Dana, planted in a production orchard at locality Hustopeče (48.9478931N, 16.7382247E) in South Moravia.

The symptoms shown by the various trees included mild leaf yellowing, mild chlorotic mosaics, reduced fruit development and irregular fruit ripening. Each tree was sampled individually; leaves were collected from different symptomatic branches and kept in the cold during the sampling and transport to the laboratory, then stored at −80 °C until RNA isolation. 

### 2.2. RNA Isolation

For sample B15, double-stranded RNAs were isolated from 10 g of pooled fresh leaves using batch chromatography on CF11 cellulose (Whatman, Maidstone, UK), as described previously [28,29]). For all other samples, total RNA was purified for each tree using 0.1 g of four pooled fresh or frozen leaves and the Plant/Fungi Total RNA purification kit (Norgen Biotek, Thorold, ON, Canada). Leaves were homogenized in double the volume of buffer recommended by the kit, using a FastPrep homogenizer. Ensuing extraction steps followed the kit manufacturer’s recommendations. Purified total RNA was cleaned up and concentrated using the RNA Clean-Up and Concentration Micro-Elute Kit (Norgen Biotek, Thorold, ON, Canada) for high-throughput library construction purposes. Purified RNAs were kept frozen at −80 °C until use.

### 2.3. High-Throughput Sequencing

A cDNA library was constructed from purified dsRNA (sample B15) using the True Seq Stranded mRNA Library preparation kit (Illumina), and sequenced in a multiplexed run on the HiSeq2500 (1 × 100 bp) platform (SEQme s.r.o., Dobříš, Czech Republic). Purified and concentrated total RNA from samples B160, B42 and B78 was ribodepleted using the QIAseq FastSelect-rRNA Plant kit (Qiagen, Hilden, Germany) according to the manufacturer´s recommendation, and cDNA libraries were prepared using the NEBNext Ultra II Directional RNA Library Prep Kit (New England Biolabs, Ipswich, MA, USA). Sequencing was then performed in a multiplex run on the NovaSeq6000 (2 × 161 bp) platform at the Sequencing Centre of the Institute of Experimental Botany of the Czech Academy of Sciences, v. v. i. and Palacký University, Olomouc (CATRIN-UPOL, Olomouc, Czech Republic).

### 2.4. High-Throughput Sequencing Data Analysis

Following quality trimming, HTS reads were de novo assembled using the Spades algorithm implemented in Geneious Prime 2024.02 ((Dotmatics, Boston, MA, USA) with default parameters. The obtained contigs were annotated against the NCBI (GenBank) virus database using the BLASTX and BLASTN algorithms [30]. Contigs of interest were extended by reads mapping using the Geneious mapper (low sensitivity, iteration 5×). Final contigs were used for the design of the primers used to complete viral genomic sequences using RACE-PCR. The genomic sequences of novel viruses and selected contigs from the B15 plant were annotated and deposited in GenBank under accession numbers PP711309–PP711325).

### 2.5. Completing Virus Sequence, RACE-PCR and Sanger Sequencing 

Nearly complete genomic sequences of the novel viruses were completed using the SMARTer RACE 5′/3′Kit (Takara Bio, San Jose, CA, USA) on total RNA for both 5′ and 3′ ends, in direct or nested PCR. For the 3′ end amplification, total RNA was adenylated using *E. coli* Poly(A) Polymerase (New England BioLabs, Ipswich, MA, USA) according to the manufacturer´s recommendations. 

All primers (Supplementary Table S1) were designed using Geneious Prime 2022.0 (Dotmatics, Boston, MA, USA) and their specificity was verified using BLASTN against HTS contigs and the GenBank database (NCBI). Amplicons were purified using the NucleoSpin Gel and PCR Clean-up kit (Macherey-Nagel, Düren, Germany) and Zymo-Spin III columns (Zymo Research, Freiburg im Breisgau, Germany) and directly Sanger-sequenced using the BigDye™ Terminator v3.1 Cycle Sequencing Kit (ThermoFisher Scientific, Waltham, MA, USA) and a Genetic Analyzer ABI Prism 3170. Due to a mixed sequence output, the RACE-PCR products obtained for sample B160 were cloned into the pGem-T plasmid, and the inserts of at least five colonies were Sanger-sequenced, as described above. The obtained Sanger sequences were analyzed and assembled with the HTS contigs to verify and complete genome ends using Geneious Prime assembler 2022.0 (Dotmatics, Boston, MA, USA).

### 2.6. RT-PCR Virus Detection

To screen for virus presence in sampled trees, RT-PCR assays were performed. The two primer pairs used were newly designed using Geneious Prime 2022.0; (1) the universal RhabF (5′-AGCAATCAATATGCGCCACC-3′) and RhabR (5′-CTTGAGGATCGCCTTTGCTG-3′) primer pair targets the *N* gene region and yields an amplicon of 479 bp, and (2) the B42 isolate-specific primer pair, B42-1f (5′-ACCTTGCTTGCTGTCACTTT-3′) and B42-1r (5′-ACCTCCTACAACCCAAGGAA-3′), targets the *M* gene region and yields an amplicon of 690 bp. Three to five hundred nanograms of purified total RNA were used to synthetize complementary DNA using random hexamers and the Bioscript reverse transcriptase (Meridian Biotek, The Woodlands, TX, USA) in a total volume of 20 µL according to the manufacturer’s recommendation. PCR amplifications were performed using MyTaq DNA polymerase (Meridian Bioline, The Woodlands, TX, USA) and 0.2 mM of each RhabF /RhabR primersin with a total volume of 25 µL. Alternatively, the B42-1f/B42-1r primer pair was used for the specific detection of the B42 isolate during transmission tests. Amplification cycle conditions, identical for both primer pairs, were as follows: 95 °C for 2 min and 35 cycles (95 °C for 1 min, 58 °C for 1 min, 72 °C for 1 min) followed by a final extension step of 72 °C for 7 min. PCR amplicons were separated by electrophoresis in 1.5% agarose gel in TAE buffer and visualized with the GelRed nucleic acid stain (Biotium, Fremont, CA, USA) and the Syngene gel documentation system. The specificity of amplicons was verified by Sanger sequencing.

### 2.7. Genetic and Phylogenetic Analyses

Open reading frames were detected using the Glimmer (Geneious Prime 2024.02) and ORFinder (NCBI) algorithms, and their position was verified by detecting rhabdovirus-specific gene junction sequences using the MEGA 11.0.11 alignment Explorer [31]. 

Multiple alignments of the obtained novel virus sequences and selected nucleorhabdovirus sequences available in GenBank were obtained with the ClustalW algorithm and used to generate nt and aa distance matrices, which were used for the following genetic and phylogenetic analyses: (1) the genetic variability and frequency of synonymous and nonsynonymous mutations, as well as the selection ratio, were assessed using p-distance, Z-test and Tajima test neutrality [31,32]; (2) potential recombination events were screened using RDP5 [33]; (3) nuclear localization signals were predicted using the Stradamus algorithm [34] available at the Novolab web page (https://www.novoprolabs.com/tools/nls-signal-prediction, accessed on 10 April 2024), using a posterior threshold value of 0.6; and (4) phylogenetic trees were constructed using the neighbor-joining method and Tamura 3-parametric and JTT models (selected using the Model algorithm) for nt and aa sequences, respectively. Bootstraps repetitions (1000×) were used for both analyses. The constructed phylogenetic trees were visualized using Tree Explorer. All analyses were performed using MEGA 11.0 [31].

### 2.8. Biological Testing–Transmission Trials

Seeds from the elderberry fruits from the infected B15 and B42 shrubs were washed, dried on filter paper at room temperature and cold-stratified in wet sand in a closed plastic bag in a refrigerator at 8 °C for two to three months. Fifty germinated seedlings were transferred to soil and grown in 9 cm diameter pots and planted in a greenhouse at a temperature of 24 °C/19 °C with a 16 h/8 h photoperiod. Plantlets were visually inspected at weekly intervals for two months from the development of the first true leaf. The presence of a virus was tested by RT-PCR, as described above, one and two months after germination.

In the transmission test, 1 g of fresh leaves collected from a virus-infected tree (B42) was homogenized in PBS extraction buffer (consisting of 137 mM NaCl, 2.7 mM KCl, 10 mM Na_2_HPO_4_, and 1.8 mM KH_2_PO_4_, pH = 7.4) at a ratio of 1:9 and 1% Celite as an abrasive. The inoculum was rub-inoculated on two leaves of 25 virus-free elderberry seedlings. Inoculated plants were visually inspected once per week over a 2-month period and the presence of the virus was checked by RT-PCR using the isolate B42-specific primer pair (B42-1f/B42-1r), as described above.

## 3. Results

During the study of the elderberry virome in the Czech Republic, one of the analyzed trees, marked as B15, was analyzed in detail by the high-throughput sequencing of purified dsRNA using the Illumina platform. A total of 27,747,697 reads were thus obtained. The relatively high proportion of viral reads allowed us to identify a complex mixed infection involving known viruses (cherry leafroll virus, elderberry carlaviruses A and B) and novel viruses (Sambucus virus S of the genus *Bromovirus*, elderberry aureusvirus 1 of the genus *Aureusvirus*, and Sambucus virus 1 of the genus *Cytorhabdovirus*) [28,35,36]. A subsequent analysis brought the surprising identification of a number of mainly shorter contigs showing in BLASTX analysis homologies with proteins of various betanucleorhabdoviruses and in particular with those of datura yellow vein virus (DYVV, YP_009176972-74, YP_009176976-77). This is exemplified by the largest of these contigs; the partial protein encoded by contig B15-15 (3553 nt, GenBank Acc. No. PP711325, corresponding to 255 reads) showed 39.2% aa identity with the RdRp of DYVV (YP_009176977), and the partial protein encoded by contig B15-6 (1968 nt, PP711322, 98 reads) showed 49.2% identity with the movement protein of green Sichuan pepper nucleorhabdovirus (YP_010797761), while those encoded by contigs B15-7 (1664 nt, PP711323, 65 reads) and B15-10 (1263 nt, PP711324, 55 reads) showed, respectively, 55.1 and 51% identity with the glycoprotein of the Bacopa monnieri virus 2 (YP_010798893).

Unfortunately, due to the presence of several viral isolates, it was not possible to further extend these contigs or to reliably assemble them in (a) potential scaffold(s) for subsequent analysis. It was thus decided to screen further elderberry plant material by RT-PCR using the RhabF/RhabR primer pair designed against the partial *N* (nucleoprotein) gene sequence of the identified virus. Three elderberry plants, originating from three different Czech localities, were thus found positive for the targeted betanucleorhabdovirus and selected for further analysis. Two of them (B160, B42) are wild growing shrubs, while the third one is a tree of cv. Dana planted in a production orchard. All of them showed only mild virus-like symptoms such as mild leaf or vein chlorosis on some branches, and also irregular flower development and fruit ripening.

High-throughput sequencing of ribodepleted total RNA, using the Ilumina platform, yielded 128,333,812 paired reads for B160: 115,658,948 for B78 and 156,466,598 for B42. BLASTX analysis of the assembled contigs against the NCBI viral database allowed for the identification of eight contigs in sample B160, four contigs in B78 and one contig in B42; all of these were approximately 13.5 kb in length (Table 1), and showed the highest encoded proteins aa sequence identity (63.8 to 64.8%) with the HWY65 isolate of sowhistle yellow vein virus 1 (NC_076510). Comparisons of the obtained putative elderberry betanucleorhabdovirus sequences with the genomic sequences of other betanucleorhabdoviruses available in GenBank confirmed the observed divergence of the assembled viral genomes from all other betanuclorhabdoviruses. A p-distance analysis performed on the ClustalW-constructed distance matrix showed that all of them share the highest nucleotide identity levels (64.4 to 65.2%) with the BER19ST2 isolate of tomato betanucleorhabdovirus 1 (OL472119), and with isolates SKO20ST2 (OL472113) and 09-50 (OP441765) of tomato betanucleorhabdovirus 2 (64.7 to 65.9%). Identity levels with all other betanucleorhabdoviruses were lower (43.2 to 53.8%). In all cases, an identity level below the ICTV species demarcation criterion in the genus (75% identity for the complete genome sequence) [2] was observed. In addition, detailed comparisons between the various elderberry isolates showed that they share from 69.4 to 98.9% nucleotide identity (Supplementary Table S2). A more detailed analysis showed that the 13 sequences can be regrouped in five clusters showing >83.7% intracluster pairwise nt identity, but between 74% and 69.4% pairwise nt identity between clusters. This observation suggests the existence of five closely related novel member species in the genus *Betanucleorhabdovirus*.

Screening using RDP5 for the presence of potential recombination events between the various elderberry viral isolates yielded negative results, despite the fact that the majority of isolates were identified in the same tree.

**Figure 1:**
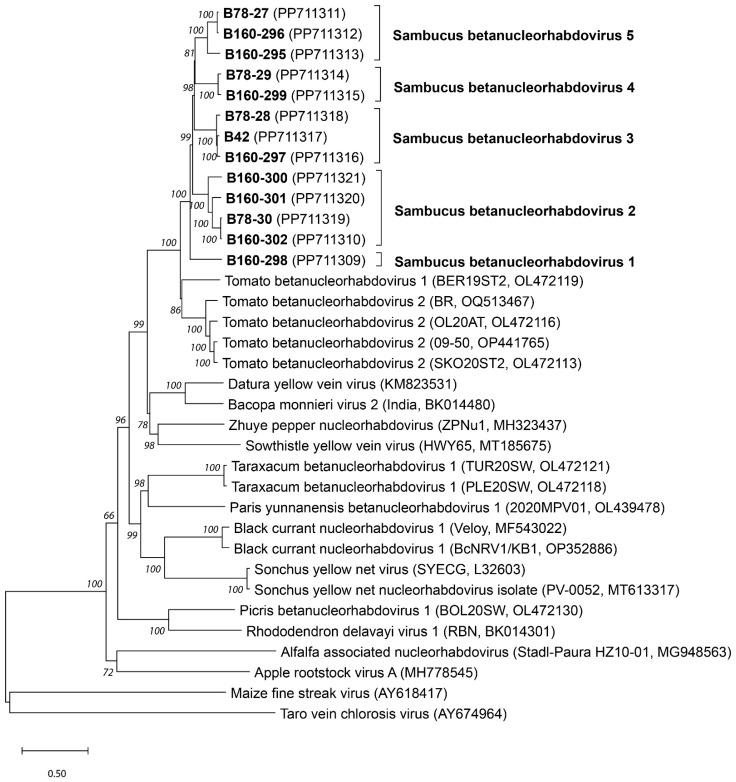
The phylogenetic tree of novel Sambucus betanucleorhabdoviruses and other betanucleorhabdoviruses representative isolates constructed based on complete genome sequences using neighbor-joining analysis and the Tamura–Nei model, with 1000× bootstrap replications. The Sambucus putative novel species are shown in bold, with isolate names and GenBank Acc. No. (in parentheses). The scale bar represents 50% nucleotide diversity; only bootstrap values ≥70 are shown. The sequences of gamanucleorhabdovirus MFSV (AY618417) and alphanucleorhabdovirus TaVCV (AY674964) were used to root the tree.

Phylogenetic analyses using neighbor-joining (Figure 1) and maximum likelihood algorithms (the ML tree had the same topology as the NJ tree, not shown) showed that all elderberry isolates group together in a fully supported cluster, away from all other betanucleorhabdoviruses. These analyses also confirmed the existence of five distinct and well-supported sub-clusters of isolates: isolate B160-298 represents the first cluster; isolates B160-300, B160-301, B160-302 and B-78-30 form the second cluster; isolates B160-297, B78-28 and B42 form cluster 3; isolates B78-29 and B160-299 form the fourth cluster and isolates B160-296, B160-295 and B78-27 form the fifth cluster (Figure 1). The names Sambucus betanucleorhabdovirus 1 to 5 are, respectively, proposed for these clusters of isolates, since the phylogenetic trees constructed using both the NJ and ML algorithms strongly support a conclusion about the existence of five novel betanucleorhabdovirus species. All elderberry virus isolates are most closely related to isolates of tomato betanucleorhabdovirus 1 and tomato betanucleorhabdovirus 2, and are clearly separated from the isolates of other *Betanucleorhabdovirus* species.

### 3.1. Genome Organization of the Sambucus Betanucleorhabdoviruses 

**Figure 2:**
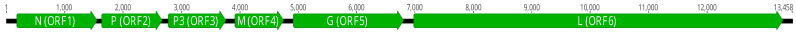
Schematic map of the Sambucus betanucleorhabdovirus genome. N—nucleoprotein, P—phosphoprotein, P3—movement protein gene, M—matrix protein, G—glycoprotein, L—large protein.

Nearly complete genomes, covering complete coding sequences, were obtained for 10 virus isolates, and complete genome sequences were obtained for three of these isolates by combining the HTS and RACE-PCR analyses. The elderberry betanucleorhabdovirus isolates show an identical genome structure with the six 3′-N-P-P3-M-G-L-5′ open reading frames (Figure 2, Table 2) separated by gene junction sequences, and surrounded by 3′ leader and 5′ trailer sequences, all typical of the negative single-strand RNA genomes of the *Rhabdoviridae* family and the *Betanucleorhabdovirus* genus, respectively. In silico analysis allowed for the identification of ORF1, ORF2, ORF3, and ORF6 with identical lengths of, respectively, 1374 nt, 1032 nt, 978 nt, and 6321 nt for all isolates. On the other hand, the various isolates differed in the length of ORF4 and ORF5, with the *G* gene being 1884 or 1890 nt, and the *M* gene being even more variable with predicted sizes of 825 nt, 834 nt, 837 nt, and 849 nt (Table 2).

**Figure 3:**
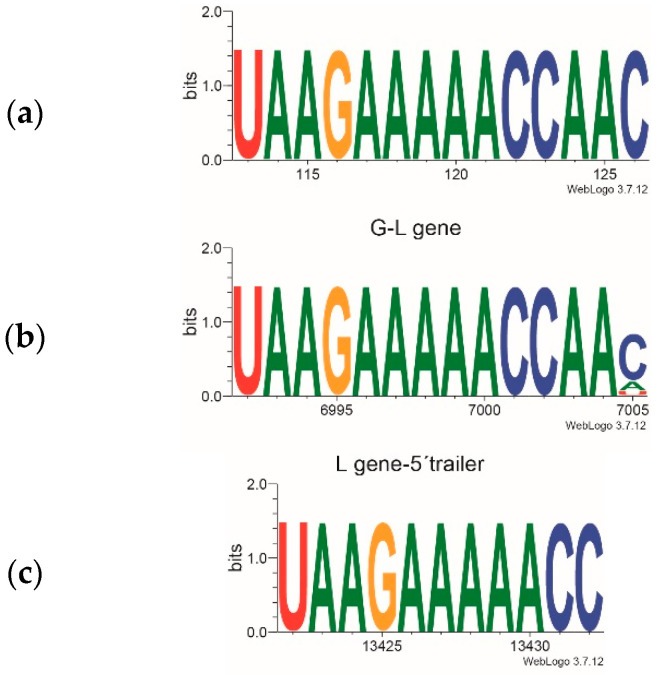
Weblogos reflecting the variability of gene junction sequences present in the genome sequences of studied Sambucus betanucleorhabdovirus isolates. Gene junction motif position is shown as follows: (**a**) common motif, (**b**) *G—L* genes motif, and (**c**) *L* gene —5´trailer.

The sequence of the detected typical rhabdovirus gene junctions localized between ORFs is highly conserved (Figure 3), with the UAAGAAAAACC motif followed by a variable AAH motif with an assumed function in transcription start [1]. An identical polyA-signal was found in all isolates at the beginning of the genome before ORF1 and at its end, after the ORF6.

#### 3.1.1. Sambucus Betanucleorhabdovirus 1

**Figure 4:**
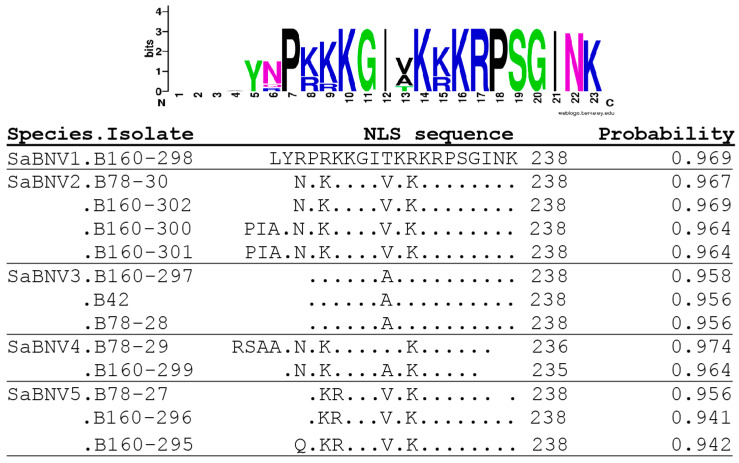
Nuclear localization signals detected in the Matrix protein in the isolates of Sambucus nucleorhabdoviruses using the Stradamus algorithm with a posterior value of 0.6.

Using RACE-PCR, a complete genome sequence was obtained for the B160-298 isolate. It is 13,488 nt long and encodes six ORFs. The ORF1, *N* gene at positions 182–1555, encodes a 457 aa long nucleoprotein (MW = 50.8 kDa) with the typical domain of the rhabdo_ncap_2 family (cl03939, pfam03216) at aa positions 149–348 (nt 445–1044). ORF2 (*P* gene) is at positions 1639–2670 and encodes a phosphoprotein of 343 aa (MW = 37.8 kDa) with a transcription factor function; ORF3 (*P3* gene, positions 2775–3752) encodes a movement protein of 325 aa (MW = 37.1 kDa); ORF4 (*M* gene, positions 3934–4767) encodes a matrix protein of 277 aa (MW = 30.5 kDa) which bears a nuclear localization signal (NLS) at aa positions 219–238 (Figure 4); and ORF5 (*G* gene, positions 4943–6826) encodes a glycoprotein of 627 aa (MW = 70.0 kDa). The largest ORF6 (*L* gene, positions 7007–13,327) encodes the viral polymerase (2106 aa, MW = 238.1 kDa) with the following expected conserved protein domains: RNA-dependent polymerase (pfam00946, aa position 214–1128) and mononegavirales mRNA-capping domain (pfam14318, aa position 1146–1372). The length of the 3´ leader sequence of the negative RNA molecule is 181 nt, and the length of the 5´ trailer sequence is 161 nt (Table 2).

The complete genome of the B160-298 isolate shows 69.4 to 70.4% nt identity with the complete or near-complete genomes of the other elderberry betanucleorhabdovirus detected in our study, and even lower identity levels with any other betanucleorhadovirus, the closest being tomato betanucleorhabdovirus 1 and 2 (65.1 to 65.2% nt identity (Table 3 and Table S2). 

#### 3.1.2. Sambucus Betanucleorhabdovirus 2

Isolates B160-300, B160-301, B160-302 and B-78-30 originated from a wild-growing elderberry shrub (B160) or from an elderberry tree of the ‘Dana’ cultivar (B78), and represent the second phylogroup of isolates, for which the name Sambucus betanucleorhabdovirus 2 is proposed. A complete genome sequence (13,458 nt) was determined for isolate B160-302 (2425× average coverage), and nearly complete genomes covering the full coding potential, as well as large parts of the leader and trailer sequences, were obtained for the three other isolates (respectively, 13,453 nt for B160-300, 13,448 nt for B160-301 and 13,438 nt for B78-30). The only difference in genome organization, as compared to Sambucus betanucleorhabdovirus 1, concerns the *M* gene, which is 825 nt long for isolates B160-302 and B78-30 and 834 nt long for isolates B160-300 and B160-301. For isolate B160-302, the length of the 3′ leader is 178 nt and that of the 5′ trailer is 158 nt, respectively (Table 2).

Sambucus betanucleorhabdovirus 2 isolates show 98.9 to 83.7% genome identity with each other, but only 71.2 to 72.3% identity with the other elderberry betanucleorhabdovirus isolates and 64.4 to 65.7% identity with tomato betanucleorhabdovirus 1 or 2 isolates (Table S2).

Three of the four longest HTS contigs detected in the B15 tree discussed above represent part of the genome of a Sambucus betanucleorhabdovirus 2 isolate since they, respectively, share 98.1% (contig B15-15, partial *L* gene), 99.8% (contigs B15-10, partial *G* gene) and 98.3% (contig B15-6, partial *M* gene) with the B160-302 isolate.

#### 3.1.3. Sambucus Betanucleorhabdovirus 3

Isolates B160-297, B78-28, and B42 belong to the Sambucus betanucleorhabdovirus 3 species. A complete genome (13,521 nt, 53.9× average coverage) was obtained for the B42 isolate, and nearly complete genomes, respectively, 13,508 nt long for B160-297 and 13,518 nt for B78-28, were obtained. The genome structure and length of ORFs are, again, identical to those of Sambucus betanucleorhabdovirus 1, except for the length of the *M* and *G* genes. The *M* gene is 837 nt long and the *G* gene is 1890 nt long. As seen in the B42 complete genome, the length of the 3′ leader is 176 nt and that of the 5′ trailer is 165 nt (Table 2).

The B160-297, B78-28, and B42 isolates show 95.3% to 96.9% genome nt identity with each other, but only 69.8 to 73.6% identity with the other elderberry betanucleorhabdovirus isolates, and 64.6 to 65.3% identity with tomato betanucleorhabdoviruses 1 and 2 (Table S2).

#### 3.1.4. Sambucus Betanucleorhabdovirus 4

Two isolates, B160-299 and B78-29, represent Sambucus betanucleorhabdovirus 4, and nearly complete genomes of, respectively, 13,486 nt and 13,482 nt were obtained for them, covering whole CDSs and lacking only short parts of the 5′ and 3′ UTRs. Both share an identical genome structure but, again, show differences with other isolates in *M* and *G* gene length, with an *M* gene of 837 nt and a *G* gene of 1890 nt. These two isolates show 96.4% genome nt identity but only 69.9 to 74.0% identity with the other elderberry betanucleorhabdovirus isolates, and 64.7 to 65.3% identity with tomato betanucleorhabdoviruses 1 and 2 (Table 2 and Table S2). 

#### 3.1.5. Sambucus Betanucleorhabdovirus 5

Three isolates, B160-295, B169-296 and B78-27, represent the last species, Sambucus betanucleorhabdovirus 5. Nearly complete sequences 13,527 nt (B160-295), 13,524 nt (B160-296) and 13,524 nt (B78-27) were obtained, covering whole CDSs and lacking only short parts of the 5′ and 3′ UTRs. While showing an identical genome structure, they differ from other isolates by having an 849 nt long *M* gene. These three isolates show 84.7 to 98.2% genome nt identity with each other, but only 70.2 to 74.0% identity with the other elderberry betanucleorhabdovirus isolates, and 64.6 to 65.3% identity with tomato betanucleorhabdoviruses 1 and 2 (Table 2 and Table S2).

The presence of a Sambucus betanucleorhabdovirus 5 isolate in the B15 tree can be deduced from the observation that one of the longest contigs (B15-7-9, partial *G* gene) shows 97.7% nt identity with the B78-27 isolate genome.

### 3.2. Genome Analysis of Sambucus Betanucleorhabdoviruses

As the discrimination of Sambucus betanucleorhabdoviruses seems to reflect variability in the length of ORF4 (Matrix protein) and ORF5 (Glycoprotein), we performed a comparative analysis of each ORF and of the encoded proteins. The isolates showed 79% to 99.5% identity of their concatenated amino acid sequences among all elderberry betanucleorhabdovirus isolates. The intraspecies and average interspecies cluster pairwise aa identities of isolates ranged, respectively, between 95.1% and 98.8% and between 70.8% and 74.9% (Table 3 and Table S2). 

**Figure 5:**
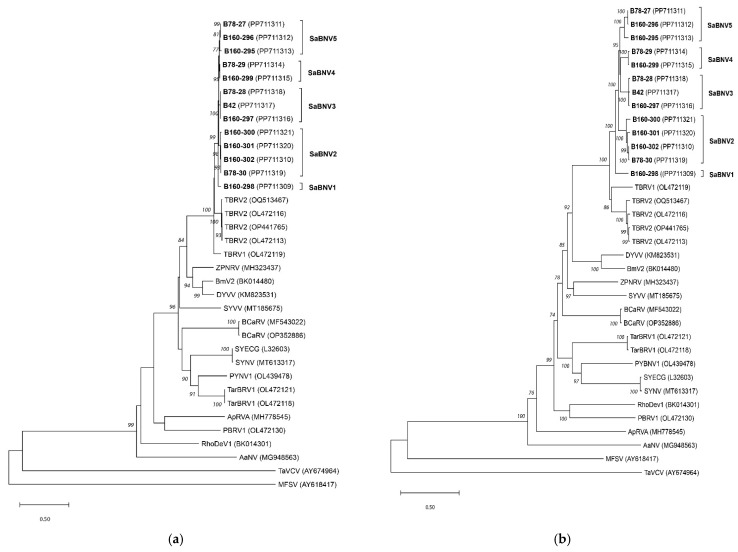
Phylogenetic trees of novel Sambucus betanucleorhabdoviruses and the other betanucleorhabdovirus-representative isolates constructed based on the nucleoprotein (**a**) and large protein (**b**) aa sequences constructed using neighbor-joining analysis and the JTT model, with 1000× bootstrap replications. The Sambucus isolates are marked in bold, betanucleorhabdovirus isolates are marked according to their assignment species identity and GenBank Acc. No. (in parenthesis), and the bar represents 50% nucleotide diversity. Only bootstrap values ≥70 are shown, and the sequences of MFSV (AY618417) and TaVCV (AY674964) were used to root the tree. The virus abbreviations are as follows: SaBNV1–5, Sambucus betanucleorhabdovirus 1–5; TBRV2, tomato betanucleorhabdovirus 2; TBRV1, tomato betanucleorhabdovirus 1; DYVV, Datura yellow vein virus; BmV2, Bacopa monieri virus 2, ZPNRV, Zhuye pepper nucleorhabdovirus 1; SYVV, sowhistle yellow vein virus; BCaRV, blackcurrant associated rhabdovirus; TarBRV1, Taraxacum betanucleorhabdovirus 1; PYNV1, Paris yunnanensis betanucleorhabdovirus 1; SYECG, Sonchus yellow net virus; SYNV, Sonchus yellow net nucleorhabdovirus; RhoDeV1, Rhododendron delavay virus 1; PBRV1, Picris betanucleorhabdovirus 1; ApRVA, apple rootstock virus A; AaNV, alpha–alpha-associated nucleorhabdovirus; MFSV, maize fine streak virus; and TaVCV, Taro vein chlorosis virus.

Nucleotide sequence pairwise identity levels for the *N* gene varied from 77.1 to 80.1% for comparisons involving isolates belonging to different clusters and between 86.2 and 98.8% for comparisons between isolates of the same cluster. A similar trend of a gap between inter-species and intraspecies comparisons was also observed for all other genes and encoded proteins (Table S2). Despite the differences in the level of variability shown by each gene, phylogenetic reconstructions performed independently using the various genes or encoded proteins yielded phylogenetic trees with a topology similar to that of the complete genome tree (only phylogenetic trees constructed on the nucleoprotein and large protein sequences are shown, Figure 5), lending support to the RDP5 analysis and the conclusion that no recombination events link the analyzed elderberry isolates. 

All genes show a high frequency of synonymous mutations over non-synonymous ones, which indicates, together with the D values calculated in the Tajima test of neutrality and negative Z test values, that relatively strong conservative negative selection pressures apply to all genes (Table 4).

### 3.3. Preliminary Results on Sambucus Betanucleorhabdoviruses Spread Mechanisms

The transmission experiments performed suggest that the Sambucus betanucleorhabdoviruses are not transmissible by seeds, as they were not detected during the repeated analysis of fifty elderberry seedlings grown from the seeds collected from infected elderberry trees (B42, B15). Attempts at the mechanical inoculation of virus-free seedlings using a crude extract from the leaves of the B42 tree were also unsuccessful, as the virus was not detected in any of the 25 inoculated plants. However, it should be considered that based on the abundance of viral reads in the HTS data, virus titer in the inoculum source used was low. Further transmission trials are in progress.

## 4. Discussion

Elderberry is a wild shrub or small tree, which is widespread in central Europe. It has a historical place in traditional folk medicine because of the antiviral and antibacterial effects of the substances present in its flowers and fruits. Moreover, elderberry is also used in the food industry for its antioxidant properties, its known positive effect on human health, and as a natural additive to improve food quality and replace artificial substances in products [20,24,37]. 

Before the use of next-generation sequencing tools, little information was available on the elderberry virome, but recent studies have brought about the description of several new RNA viruses infecting elderberry and belonging to different viral families and genuses, i.e., elderberry carlaviruses, Sambucus virus S (genus *Bromovirus*), elderberry aureusvirus 1 and Sambucus virus 1 [28,35,36,38]. Here, isolates of new plant betanucleorhabdoviruses were detected in HTS data and their presence was repeatedly confirmed in the original tree, as well as in other wild-growing or production orchard elderberry trees.

The characterized viral isolates cluster in five distinct phylogroups that fulfill the currently accepted 75% nt whole-genome divergence demarcation criterion, suggesting that these isolates belong to five novel species within genus *Betanucleorhabdovirus.* However, it should be noted that besides the 75% identity criterion, the ICTV also notes that species within the genus Betanucleorhabdovirus “occupy different ecological niches as evidenced by differences in hosts and/or arthropod vectors” and “can be clearly distinguished in serological tests or by nucleic acid hybridisation”. Given that they were all identified in elderberry and that their vector(s) is(are) not known, it is difficult to assess whether the isolates analyzed here meet the second criterion set forth by the ICTV. Likewise, in the absence of further experimental work, it will not be possible to evaluate whether the third criterion is met. The analysis of the taxonomic status of the isolates analyzed here thus highlights one of the current issues in viral taxonomy, the relative weight to be given to molecular criteria over other biological ones. 

A general feature of high-throughput sequencing is that this approach allows for the detection of a wide range of novel viruses, but their description is quite often based only on genetic data, without or with only limited knowledge about their biology [39,40,41]. A choice must then be made between giving priority to molecular criteria and using available genome data to solve the question of the taxonomic status of novel agents, or giving priority to unavailable biological parameters and thereby delay, possibly forever, a decision on taxonomic status.

It is interesting to note that the situation reported here for elderberry betanucleorhabdoviruses parallels that of elderberry carlaviruses A to E (ElVA-E), for which the genetic demarcation criterion was fulfilled absolutely or partially, resulting in the proposed elderberry carlavirus A and B species still remaining unclassified [38,42]. In both cases, a group of closely related isolates at, or close to, the molecular species discrimination criteria were observed in elderberry, suggesting an ongoing radiative speciation mechanism. In this respect, it is interesting to notice that despite having been identified in different trees sampled in different regions, the isolates reported here do not show a continuum of pairwise divergence values, but rather a bimodal distribution of these values cleanly separated at the currently accepted 75% species demarcation value. This suggests that this speciation mechanism is already well advanced enough that the intraspecies variability that has accumulated posterior to the isolation of viral lineages is clearly lower than the genetic distance separating the species. It is, of course, tempting to speculate on the evolutionary forces or constraints that may have contributed to this speciation burst in the case of elderberry carlaviruses and betanucleorhabdovirus, and on why such speciation events do not seem to have affected other elderberry viruses. In any case, we suggest that the clear bimodal nature of the pairwise identity values and the fit with the 75% demarcation criterion strongly argue in favor of considering the existence of five distinct species, despite the fact that they share the same host and, possibly, the same vector(s). While this(ese) vector(s) remain unknown, the phylogenetic position of the elderberry isolates suggests that they are transmitted by aphids, with species such as *Aphis sambuci* or polyphaguous *A. horii* and *Myzus persicae* as potential candidates.

Although betanucleorhabdovirus infections are often associated with symptoms of vein clearing and yellow vein mosaics [5,19,43], the infected plants analyzed here showed either no symptoms or only mild leaf chlorosis, as well as irregular fruit ripening, irregularly distributed over the canopy. However, the symptoms observed could be caused by the other viruses present in mixed infection in the analyzed plants, such as CLRV, or they could be the result of the synergic interaction of mixed virus infection by elderberry carlaviruses, elderberry aureusvirus 1 or Sambucus virus 1, detected in the same plants [28,35,36]. Further studies on the genetic variability, epidemiology and geographical distribution of elderberry betanucleorhabdoviruses are necessary to deepen knowledge about the elderberry virome and to elucidate their importance in the health status of the elderberries.

## Figures and Tables

**Table 1:** Characteristics of the contigs obtained for the Sambucus betanucleorhabdovirus isolates from three *Sambucus* samples.

Isolate (Putative Species)	Sequence Length	Betanucleorhabdovirus Reads (% of Total #)	Reads Coverage
B160-295 (SaBNV5)	13,527	22,782 (0.018%)	227.4
B160-296 (SaBNV5)	13,524	64,643 (0.050%)	647.9
B160-297 (SaBNV3)	13,508	88,653 (0.069%)	892.1
B160-298 ^cg^ (SaBNV1)	13,488	570,049 (0.444%)	5770.2
B160-299 (SaBNV4)	13,486	87,614 (0.068%)	883.8
B160-300 (SaBNV2)	13,453	43,583 (0.034%)	441.3
B160-301 (SaBNV2)	13,448	293,265 (0.229%)	2962.1
B160-302 ^cg^ (SaBNV2)	13,458	240,413 (0.187%)	2425.2
B78-27 (SaBNV5)	13,524	269,171 (0.233%)	2824.0
B78-28 (SaBNV3)	13,518	268,699 (0.232%)	3141.6
B78-29 (SaBNV4)	13,482	323,456 (0.280%)	3426.7
B78-30 (SaBNV2)	13,438	13,584 (0.012%)	143.4
B42 ^cg^ (SaBNV3)	13,521	4995 (0.003%)	53.9

^cg^—complete genome; SaBNV1–5—putative novel species, Sambucus betanucleorhabdovirus 1 to 5.

**Table 2:** The annotation of ORFs detected in the genome sequences of the studied betanucleorhabdovirus isolates.

	N Gene		P Gene		P3 Gene		M Gene		G Gene		L Gene		Total Length
Isolate	nt Position	Length	nt Position	Length	nt Position	Length	nt Position	Length	nt Position	Length	nt Position	Length	
**B160-298**	182–1555	1374	1639–2670	1032	2775–3752	978	3934–4767	834	4943–6826	1884	7007–13,327	6321	13,488
**B78-30**	168–1541	1374	1623–2654	1032	2765–3742	978	3909–4733	825	4906–6789	1884	6969–13,289	6321	13,438
**B160-302**	179–1552		1634–2665		2776–3753		3920–4744		4917–6800		6980–13,300		13,458
**B160-300**	174–1547		1629–2660		2770–3747		3914–4747	834	4911–6794		6975–13,295		13,453
**B160-301**	173–1546		1628–2659		2770–3747		3914–4747		4900–6783		6964–13,284		13,448
**B160-297**	171–1544	1374	1627–2658	1032	2777–3754	978	3962–4798	837	4968–6851	1884	7030–13,350	6321	13,508
**B42**	177–1550		1633–2664		2783–3760		3968–4804		4974–6857		7036–13,356		13,521
**B78-28**	171–1544		1627–2658		2779–3756		3964–4800		4970–6853		7032–13,352		13,518
**B78-29**	170–1543	1374	1626–2657	1032	2780–3757	978	3940–4776	837	4939–6828	1890	7006–13,326	6321	13,482
**B160-299**	170–1543		1626–2657		2780–3757		3940–4776		4942–6831		7010–13,330		13,486
**B78-27**	171–1544	1374	1631–2662	1032	2796–3773	978	3963–4811	849	4969–6858	1890	7040–13,360	6321	13,524
**B160-296**	171–1544		1631–2662		2796–3773		3963–4811		4969–6858		7040–13,360		13,524
**B160-295**	170–1543		1630–2661		2790–3767		3957–4805		4963–6852		7041–13,361		13,527

**Table 3:** Identity of complete nucleotide and concatenated (CDS) amino acid sequences [nt/aa%] of Sambucus betanucleorhabdovirus isolates with other betanucleorhabdoviruses selected for their phylogenetic relatedness.

		B160-298 (PP711309)	TBRV1 (OL472119)	TBRV2 (OL441765)	DYVV (KM823531)	BmV2 (BK014480)	ZPNRV (MH323437)	SYVV (MT185675)
**1**	**B160-298**	n/c	64.7/70.4	65.1/70.2	53.3/48.4	52.9/48.1	52.7/48.0	50.5/45.5
**2**	**B78-30**	69.5/79.4	64.4/69.7	65.6/70.2	52.9/48.0	53.3/48.3	52.7/48.3	50.6/45.3
	**B160-302**	69.4/79.5	64.4/69.8	65.5/70.2	53.1/48.1	53.3/48.3	52.8/48.2	50.7/45.4
	**B160-301**	69.7/79.3	64.6/69.7	65.9/70.0	53.1/48.0	53.1/48.5	52.9/48.1	50.7/45.3
	**B160-300**	69.8/79.0	64.6/70.2	65.3/70.3	53.4/47.7	53.0/48.2	52.8/48.0	50.4/44.9
**3**	**B160-297**	69.8/79.5	65.0/70.5	65.0/70.4	53.7/48.4	53.4/48.2	53.1/48.4	50.8/45.2
	**B42**	70.0/79.4	65.2/70.4	65.2/70.3	53.7/48.4	53.4/48.2	53.1/48.4	50.8/45.2
	**B78-28**	69.9/79.5	65.1/70.6	64.8/70.4	53.8/48.4	53.4/48.1	53.0/48.5	50.6/45.2
**4**	**B78-29**	70.0/80.0	64.8/70.6	65.2/70.7	53.3/48.5	53.8/48.7	52.7/48.4	50.5/45.4
	**B160-299**	69.9/79.9	64.7/70.4	65.3/70.6	53.3/48.5	53.8/48.7	52.8/48.4	50.7/45.5
**5**	**B78-27**	70.3/81.0	65.2/70.2	65.2/70.2	53.2/48.3	53.3/48.2	52.6/48.4	50.3/45.3
	**B160-296**	70.4/80.9	65.2/70.3	65.3/70.2	53.3/48.3	53.4/48.1	52.8/48.4	50.2/45.3
	**B160-295**	70.2/80.4	65.2/70.5	65.1/70.0	53.2/48.2	53.0/48.2	52.6/48.6	49.9/45.3

Virus isolates are described by their acronym and GenBank Accession No. (in parenthesis): 1–5—Sambucus betanucleorhabdovirus 1–5 species, TBRV1—tomato betanucleorhabdovirus 1, TBRV2—tomato betanucleorhabdovirus 2, DYVV—datura yellow vein virus, BmV2—Bacopa monnieri virus 2, ZPNRV—Zhuye pepper nucleorhabdovirus, SYVV—sowthistle yellow vein virus.

**Table 4:** Evaluation of group mean distances based on the coding nucleotide sequences and the potential selection effect.

dS/dN	1	2	3	4	5	All	*Ps*	*π*	*D*
***N***	n/c	0.431/0.008	0.142/0.002	0.108/0.002	0.366/0.007	0.726/0.029	0.107	0.040	0.754
***P***	n/c	0.393/0.052	0.156/0.0181	0.741/0.153	0.546/0.095	0.694/0.148	0.351	0.188	3.039
***P3***	n/c	0.424/0.006	0.138/0.001	0.148/0.001	0.323/0.008	0.717/0.037	0.122	0.047	0.888
***M***	n/c	0.375/0.024	0.148/0.004	0.115/0.013	0.309/0.017	0.672/0.108	0.319	0.147	1.955
***G***	n/c	0.429/0.024	0.142/0.006	0.113/0.004	0.378/0.024	0.718/0.096	0.311	0.148	2.181
***L***	n/c	0.413/0.026	0.144/0.009	0.122/0.006	0.365/0.025	0.510/0.110	0.472	0.233	2.446
**CDS**	n/c	0.393/0.052	0.156/0.018	0.100/0.006	0.403/0.045	0.694/0.148	0.292	0.136	2.076

Abbreviations: 1–5—Sambucus betanucleorhabdovirus species 1 to 5, n/c—non calculated, *Ps*—Number of segregating sites/total number of sites = S/n, π = nucleotide diversity, *D*—Tajima test of neutrality statistic; *N*—Nucleoprotein gene, *P*—Phosphoprotein gene, *P3*—Movement protein gene, *M*—Matrix protein gene, *G*—Glycoprotein gene, *L*—Large protein gene, CDS—coding sequences.

## Data Availability

The data presented in this study are available in the public database GenBank under Accession Numbers: PP711309-PP711325.

## Supplementary Materials

The following supporting information can be downloaded at: https://www.mdpi.com/article/10.3390/pathogens13060445/s1, Table S1: List of primers; Table S2: Sequence comparisons of novel Sambucus betanucleorhabdoviruses 1 to 5 with selected isolates of member species of genus *Betanucleorhabdovirus*.
