# Supplementary material for: Novel Betanucleorhabdoviruses Infecting Elderberry (Sambucus nigra L.): Genome Characterization and Genetic Variability
Source: Pathogens. 2024 May 24;13(6):445. doi: 10.3390/pathogens13060445 (PMC11206775; doi:10.3390/pathogens13060445)
Supplement: Supplementary file 1 [file pathogens-13-00445-s001.zip › TableS1.pdf]

Table S1: List of the primers used in RACE-PCR

name	5´-sequence-3´	use
SBNRB-Race5.1	TGGACAGTGGGTTCATTGGGGGAGGA	direct
SBNRB-Race5.2	AGGAAGGTATGTATATAAGAAAAACC	direct or nested
SBNRB-Race3.1	CGGCGCACAATTGGGTTAGGGGCC	direct
SBNRB-Race3.2	CGGCGCACAATTGGGTTAGGGGCCAAG	direct
